# A 30-year trend of dairy consumption and its determinants among income groups in Iranian households

**DOI:** 10.3389/fpubh.2024.1261293

**Published:** 2024-02-15

**Authors:** Roshanak Roustaee, Hassan Eini-Zinab, Delaram Ghodsi, Elham Mehrparvar Hosseini, Nasrin Omidvar, Hedayat Hosseini, Seyed Omid Hosseini Mousavi, Hamed Rafiee

**Affiliations:** ^1^Department of Community Nutrition, Faulty of Nutrition Sciences and Food Technology, National Nutrition and Food Technology Research Institute, Shahid Beheshti University of Medical Sciences, Tehran, Iran; ^2^Department of Nutrition Research, Faculty of Nutrition Sciences and Food Technology, National Nutrition and Food Technology Research Institute, Shahid Beheshti University of Medical Sciences, Tehran, Iran; ^3^Department of Agricultural Economics, University of Tehran, Tehran, Iran; ^4^Department of Food Science and Technology, Faculty of Nutrition Sciences and Food Technology, National Nutrition and Food Technology Research Institute, Shahid Beheshti University of Medical Sciences, Tehran, Iran

**Keywords:** dairy consumption, household, determinants, policy, sanction, *per capita*, panel analysis

## Abstract

**Introduction:**

Milk and dairy products provide essential nutrients and have the potential to prevent chronic diseases, thus reducing healthcare costs. However, there is a lack of consistent and updated data on dairy consumption trends in Iran. This study aims to analyze the trends in dairy consumption among Iranian households from 1991 to 2021, focusing on household-level determinants across different expense groups.

**Methods:**

The study uses data from the Iranian Household Expenditure and Income Survey conducted annually from 1991 to 2021 to analyze households’ dairy consumption. The data includes values and expenses of food and non-food items purchased in the previous month, as well as demographic characteristics of household members. The households were categorized into 10 deciles based on their gross expense. The econometric model used weighted mean *per capita* milk, yogurt, cheese, and total dairy consumption based on milk equivalent for each decile. The model takes into account changes in income, prices, household composition, education level, occupation, and residency area using panel data. Data preparation and model estimation were performed using RStudio and STATA17 software.

**Results:**

Based on the findings, in 1991, *per capita* milk, yogurt, and cheese consumption were 26.77 kg, 16.63 kg, and 2.42 kg, respectively. By 2021, these figures changed to 22.68 kg, 11.06 kg, and 3.79 kg, reflecting a decrease in milk and yogurt consumption but an increase in cheese consumption. Family size was positively correlated with yogurt consumption and head of the household spouse’s job score were positively correlated with milk, yogurt and cheese consumption. Also, the presence of under five-year-old children and older adults members (over the age of 60) in the household was inversely related with yogurt and cheese consumption. Female-headed households tended to purchase more cheese, while their milk purchase level was significantly lower. Residing in urban areas was negatively related to milk, while cheese and total dairy consumption was higher in urban areas.

**Discussion:**

The findings highlight the importance of targeted dairy subsidy interventions and educational programs to improve dairy consumption in Iranian households, especially among vulnerable groups. This will require urging policymakers and food system stakeholders for effective strategies that address macro-level factors to promote dairy consumption.

## 1 Introduction

Over the past 8,000 years, milk and fermented dairy products have been an integral part of the human diet, with their consumption deeply rooted in animal domestication (1, 2). Dairy products are rich sources of essential proteins and minerals, including calcium, potassium, zinc, and phosphorus, providing more micronutrients per kilocalorie than any other natural food in human diets (3, 4). In low and lower-middle-income countries where access to animal-source foods is limited, milk and dairy products play a vital role in meeting the community’s nutritional needs. Despite the challenge of determining the precise impact of each dairy product on human health, current dietary guidelines emphasize daily consumption of milk and dairy products as part of a healthy and balanced diet (5). Furthermore, this food group can potentially reduce the burden of prevalent chronic diseases and significantly lower the medical costs for the community (6).

As global data is insufficient to provide a universal recommendation for milk and dairy product consumption; countries should develop their own guidelines, taking into account various factors, including physical access, price, nutritional status, and food habits of their community. The first (2006) and second (2015) Iranian food-based Dietary Guidelines, recommend consuming 2–3 servings per day of milk or dairy products, with each serving equivalent to 250 cc of milk or yogurt or 45–60 grams of cheese and based on these guidelines, an individual requires 500 to 750 milliliters of milk or its equivalent which amounts to 182.5 to 273.75 kilograms, annually. The average dairy and milk requirement is approximately 228 kilograms per year. Additionally, the “Desirable Food Basket for the Iranian Population” recommends the daily consumption of 250 grams of dairy products (91 kilograms per year) (7). Two national surveys conducted in Iran in 1993 and 2004 provide insight into household food consumption and nutritional status. In 1993, the average *per capita* consumption of milk, yogurt, and cheese was 34 ± 3.7 g/day, 72 ± 4.5 g/day, and 11 ± 1 g/day, respectively. The *per capita* consumption of dairy products in urban and rural areas was 120 ± 8.4 g/day and 147 ± 13.7 g/day, respectively, with a national average of 131 ± 7.9 g/day (equivalent to approximately 47.8 kg of dairy products per year) (8). According to FAO data this year, Iran’s milk equivalent supply (excluding butter) was 58.84 kg/capita/year, compared to the world average of 74.03 kg/capita/year. In 2004, the average *per capita* consumption of milk, yogurt, and cheese increased slightly to 38 ± 1 g/day, 73 ± 1 g/day, and 15 ± 1 g/day, respectively. The *per capita* consumption of dairy products in urban areas (142 ± 1.8 g/day) was higher than in rural areas (134 ± 2.7 g/day) and the national average was 139 ± 1.5 g/day (equivalent to approximately 50 kg of dairy products per year) (9). At this time, FAO reported the *per capita* supply of milk as milk equivalent in Iran and the world as 63.95 kg/capita/year and 81.83 kg/capita/year, respectively.

A study conducted in 2014, using the Household Expenditure and Income Survey data revealed that *per capita* milk consumption, as the most commonly consumed dairy product, was 28.95 and 38.44 kilograms in urban and rural areas, respectively, falling far short of the recommended amounts (10). The available data shows that the consumption of dairy products in Iran is significantly lower than the recommended levels, particularly among lower socioeconomic groups. As it has been demonstrated that the intake of protein and calcium in the lowest tertile of socioeconomic status was significantly lower than in the highest socioeconomic status group (11).

Dairy consumption has the potential to reduce the burden of common chronic diseases in the population and significantly reduce healthcare costs for the community. In a study conducted by Javanbakht et al., the cost of medical expenses resulting from preventing type 2 diabetes and cardiovascular diseases can be avoided through daily consumption of 3 servings of dairy in the entire population of Iran has been estimated. The estimated costs for time intervals of 1, 5, 10, and 20 years are as follows: 83.33 million dollars, 31.661 million dollars, 21.138 million dollars, and 63.14934 million dollars, respectively (12). Thus, reducing dairy consumption may lead to increased costs related to the treatment of diseases, disability and care for patients and the older adults, and costs for lost years. For example, in Iran, the average hospitalization costs for older adult patients with osteoporosis in 2017 were estimated to be $3794.13, which should also account for the costs of disability, care for patients and costs for lost years (13). Additionally, it is important to consider dairy alternative foods in the diets of individuals who decide to reduce dairy intake, as this will involve substituting food and nutrients. For instance, replacing Saturated Fatty Acids (SFAs) in the diet with refined carbohydrates such as sugars and starches may increase the risk of Coronary Heart Disease (CHD) (14). Meanwhile, a study analyzing Iranian food intakes based on the desired food basket indicates that the quality of the Iranian diet has decreased in the past decade, resulting in decreased *per capita* consumption of milk and dairy products, meats (except for poultry and eggs), legumes, and vegetables in 2017 compared to 2011. In both periods, *per capita* consumption was less than the recommended amounts of the desired food basket (15). In such circumstances, the consumption of dairy products as an important source of valuable protein and calcium becomes doubly important.

Several factors, including demographic, social, cultural, and environmental factors, influence food choices and dietary patterns, including milk and dairy consumption. At the individual level, factors such as age, sex, education level, income, health status, nutritional knowledge, and psychological aspects, including attitudes toward foods and health, motivations, and values, affect milk and dairy consumption (16). Appendix 1, provides a brief overview of several studies that have examined the individual factors influencing dairy consumption. Beyond the individual level, social and cultural determinants such as lifestyle, societal and family norms, social pressure, social class, social network, and race/ethnicity also influence food choice behavior. Moreover, the local environment, including marketing and advertising pressures, plays a significant role in shaping the entire milk and dairy products system from production to consumption (16). Studies have shown that subjective norms and social groups, including family, relatives, co-workers/colleagues, and other individuals with whom consumers associate, played a crucial role in influencing Iranian consumers’ choice of dairy products (17, 18). It is important to note that Iran’s economy has undergone several years of double-digit inflation and economic shocks, which are likely to have had an impact on food prices and household consumption, particularly among lower socioeconomic level households. However, the exact magnitude and severity of these changes remain unclear due to the lack of valid and up-to-date data. For this purpose, various data sources such as Food Balance Sheets, Household Budget Surveys (HBS), or Individual Dietary Surveys (IDS) may be utilized, each with its own advantages and limitations. Typically, national statistical offices conduct HBS to gather nationally representative data on household expenditure, including food, primarily to construct cost-of-living indicators. While HBS data includes information on the quantities of different types of food purchased and their consumption, it represents the food provided at the household level and is often used as a proxy to estimate household food consumption (19). Given that studies on dairy consumption in Iran are generally cross-sectional and have limited sample sizes, the objective of this study is to assess the trends in dairy consumption among Iranian households from 1991 to 2021 and explore household-level determinants. In addition to understanding the current situation, identifying these determinants is necessary to develop and implement appropriate, evidence-based policies and programs aimed at reversing the declining trend in dairy consumption.

## 2 Materials and methods

### 2.1 Data preparation

This panel study utilized data derived from the Iranian Household Expenditure and Income Survey which is conducted annually between 1991 and 2021 by the Statistical Center of Iran. The data include households’ food and non-food expenses in urban and rural areas of Iran. The food expenses database contains information on 223 food items purchased by households during the previous month, including their amounts, prices, and expenses. Additionally, demographic characteristics, including age, gender, education level, occupation, and marital status of household members are also collected.

To determine households’ total dairy consumption as the dependent variable, data was extracted from households that had consumed at least one dairy item each year. The dairy items included in this study were pasteurized and non-pasteurized milk, dried milk, cream, pasteurized and non-pasteurized yogurt, non-pasteurized doogh (yogurt drink), pasteurized and non-pasteurized cheese, and keshk until 2013. From 2014 onwards, some changes were applied to the questionnaires, and other types of milk, pasteurized cream, mixed yogurt, pizza cheese, and mixed cheese were added to the survey. Butter and ice cream were excluded since butter is regarded as fat from a nutrition perspective, and for ice cream only expense data was available. All monthly amounts of dairy items purchased were converted to kilograms per year by multiplying by 12. To calculate the total amount of dairy consumed, it is not sufficient to simply sum up the values of different dairy products. Instead, we employed a conversion method that involved determining the average milk equivalents for each dairy item. This conversion represents the amount of milk required to produce a given dairy product. To establish accurate conversion coefficients for dairy products into milk, we considered the variability in production coefficients based on factors such as the characteristics of the milk consumed and the technology employed in production. To ensure the reliability of these coefficients, we sought the expertise of both our research team and a panel of experts from the Iranian Dairy Association Society. The resulting conversion coefficients, which account for these factors, are presented in Table 1. Non-consumer households were assigned a value of zero for their dairy consumption. Family size for each household was also extracted, and *per capita* consumption was calculated by dividing the consumption value by the family size. Total household expenses were calculated by summing all expense categories, and households were categorized into 10 deciles each year based on their gross expenses. The weight of each household in the sample was provided by the Statistical Center of Iran relative to the population each year. Finally, the weighted mean *per capita* milk, yogurt, cheese, and total dairy consumption for each decile was calculated and entered into the model. All data extraction and preparation were performed using Rstudio software, version 3.5.1.

**Table 1:** Conversion coefficients of dairy products into milk.

Product	Coefficient	Product	Coefficient
Pasteurized milk	1.01	UF cheese	5.5
Dried milk	11	Mixed cheese	5.5
Yogurt	1.11	Pizza cheese	4.5
Mixed yogurt	0.9	Doogh	0.5
Brine cheese	7.5	Keshk	2

### 2.2 Econometric model

The present study applies consumer demand theory to identify factors that influence the demand for dairy foods among Iranian households. The theory implies that changes in food demand result from changes in income and prices, which can be influenced by policies (20). In Iran, due to the increasing inflation rate, prices of food and non-food commodities have been continuously raised (21), and household real income is also affected by several exchange rate shocks (22). As a result, households’ food demand has changed considerably; depending on the household expense deciles. For example, *per capita* dairy consumption in the 10th decile of household expenditure has been 3.7 and 2.7 times more than the first decile in 2010 and 2011, respectively (23).

Studies have also shown that Iranian consumers prefer milk, yogurt, and cheese to other dairy items, and these items have the highest consumption among the dairy food group (9, 24). However since the same items show different price elasticity (25, 26), separate equations were used to estimate for household per-capita milk, yogurt, cheese, and total dairy intakes.

Family structure and composition, i.e., age and gender of family members, can also affect their dairy food preferences and consumption. Additionally, household size, occupation, and education are considered some of the other determinants of food choice at the household level (24, 27–31). The variables that entered the model and their definitions are shown in Table 2.

**Table 2:** The variables entered the model as determinants of household dairy consumption and their definition.

Variable	Definition
Price	Weighted mean of milk, yogurt, and cheese prices
Sex of Household’s head	As the weighted ratio of women-headed households
Presence of children under 5 years old in the family	As the weighted ratio of households with children under 5 years old
Presence of adults over 60 years old in the family	As the weighted ratio of households with adults over years old
Household education score	Weighted mean of education scores of adult members. Education scores were: 0 = illiterate, 6 = elementary school, 11 = high school and diploma, 14 = under-graduation university degrees, and 18 = graduate degrees
Mothers’ occupation	Weighted mean of mothers’ job scores. Scores were: 0 = housewives, 5 = unemployed but have income and 10 = employed
Residency area	The weighted ratio of the urban population

This study used panel data to estimate the econometrics model for assessing dairy demand function. To avoid false regression in estimating the model, the stationarity test of Levin, Lin & Chui (LLC) was used (32) and it was confirmed that some variables are integrated in the first level. Therefore, no risk of false regression was seen in the variables used in this model.

The baseline regression model is as follows:

$$Yit=\beta 0+\beta 1\;C_{\mathrm{it}}+\beta 2D_{\mathrm{it}}+\beta 3E_{\mathrm{it}}+\beta 4\;U_{\mathrm{it}}+\beta 5P_{\mathrm{it}}+\varepsilon_{\mathrm{it}}$$

In the above equation, Y indicates the weighted mean of *per capita* milk, yogurt, cheese, and total dairy consumption in household expense decile of *i* in year *t*, C*it* is a vector of family composition, D_it_ is other demographic characteristics of households, E*it* is households’ economic characteristics, U is urban residence P_it_ is the price of the product and ε _it_ is residual. Regressions were estimated by STATA17 software.

### 2.3 Ethical considerations

This study protocol was approved by the ethics committee of the National Nutrition and Food Technology Research Institute (No IR.SBMU.nnftri.Rec.1400.059).

## 3 Results

**Figure 1:**
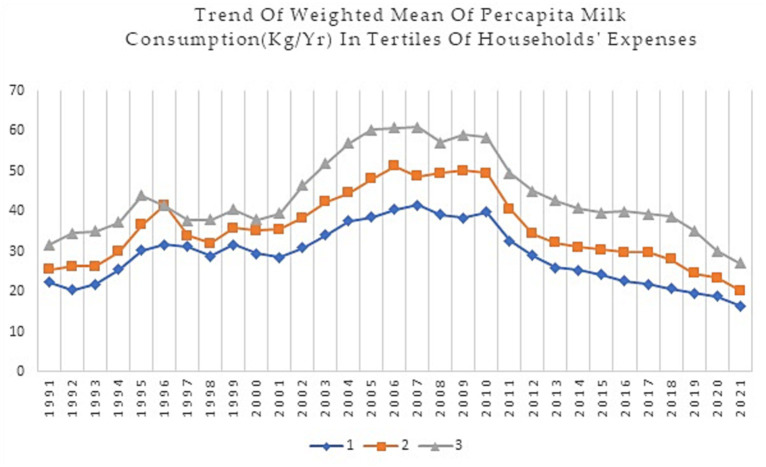
Trend of weighted mean *per capita* consumption (Kg/Yr) of milk in households’ expenses tertiles during 1991–2021.

**Figure 2:**
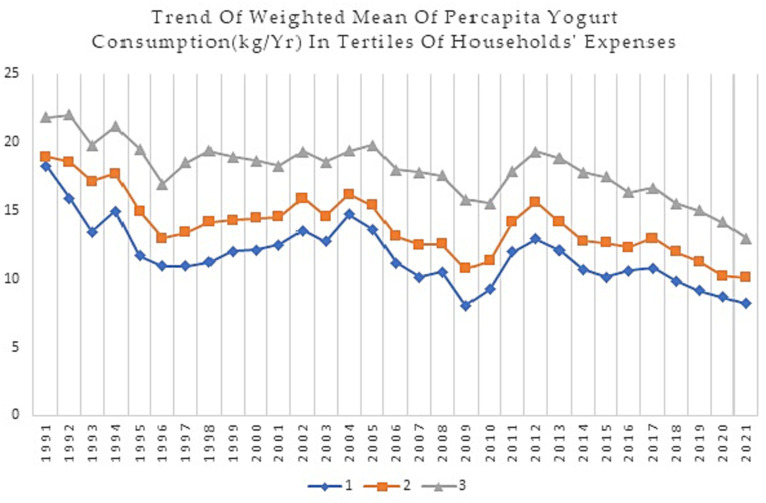
Trend of weighted mean *per capita* yogurt consumption (Kg/Yr) in tertiles of households’ expenses during 1991–2021.

**Figure 3:**
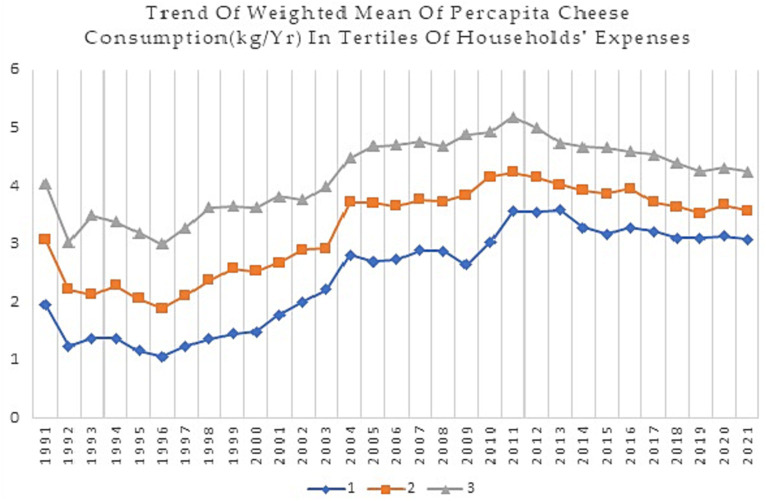
Trend of weighted mean *per capita* cheese consumption (Kg/Yr) in tertiles of households’ expenses during 1991–2021.

**Figure 4:**
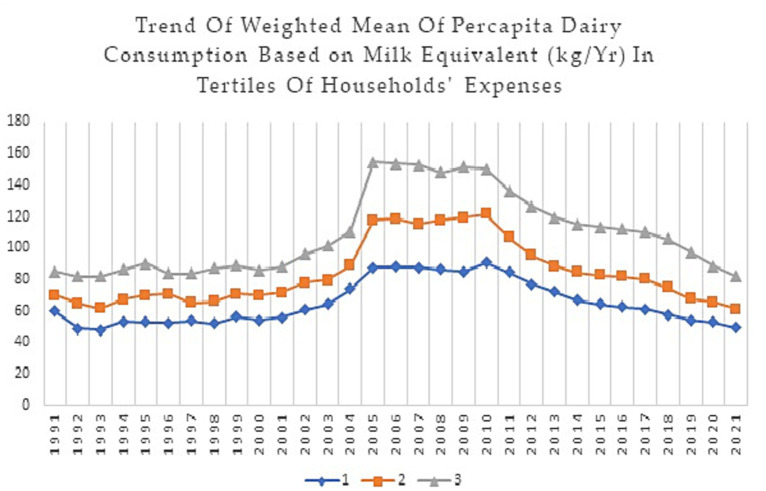
Trend of the weighted mean of *per capita* dairy consumption based on milk equivalent (Kg/Yr) in tertiles of households’ expenses during 1991–2021.

This study analyzed data on dairy food group consumption of Iranian households from 1991 to 2021. Figures 1–4 show weighted means of *per capita* milk, yogurt, cheese, and total dairy consumption, among Iranian households over thirty years. For clarity and a better understanding of the trends, findings are presented in tertiles instead of deciles.

According to Figure 1, higher-income households consume more milk and dairy products than lower-income households. There has been an overall decreasing trend in milk consumption across all tertile groups over time, although the rate of decrease varies somewhat between the income groups. In 2021, the mean milk consumption for the 3rd tertile was 27.05 kg *per capita*, as opposed to 16.50 kg *per capita* in the 1st tertile in the same year. There have also been some fluctuations in mean milk consumption; for instance, there was a slight increase in consumption in the early 2000s followed by a sharp decrease in the late 2000s and another decrease in 2010. The increasing slope of the 3rd tertile was more pronounced between 2000–2005, and the decreasing slope of consumption was slower in the 3rd tertile around 2012 compared to the other two tertiles. However, the decreasing trend in milk consumption has accelerated in both the 2nd and 3rd tertiles since 2018.

Figure 2, depicts a declining trend in yogurt consumption across all income groups, with the weighted mean of yogurt consumption dropping from 16.63 kg *per capita* in 1991 to 11.06 kg *per capita* in 2021. In terms of yogurt consumption, there was a decreasing trend until 1996, followed by a slight increase up to 2004 in the 1st and 2nd tertiles and up to 2005 in the 3rd tertile. However, there is a drop in consumption again, reaching the lowest level for the first three deciles in 2009, with a lower slope in the 3rd tertile. There has been an increasing trend since then, until 2012, when a decrease started and has continued since then.

Higher-income households tend to consume more yogurt than lower-income. Over time, the gap between the mean yogurt consumption for the 1st and 3rd tertiles has increased somewhat, from a mean difference of 2.9 kg in 1991 to 4.79 kg in 2021.

Based on Figure 3, despite the decreasing trend in *per capita* cheese consumption since 2012 for the 2nd and 3rd tertiles and 2014 for the 1st tertile, which has continued until 2021, *per capita* cheese consumption has increased over time across all tertiles of households’ expense. For example, in 1991, the *per capita* cheese consumption for the 1st tertile was 1.96, while in 2021, it increased to 3.08. Similarly, the *per capita* cheese consumption for the 3rd tertile was 4.05 in 1991 and 4.25 in 2021.

In terms of differences in cheese consumption across different tertiles, the data indicate that higher-income households tend to consume more cheese than their lower-income counterparts. For example, the 10th decile had the highest *per capita* cheese consumption in most years, while the 1st decile had the lowest *per capita* cheese consumption. The increasing trend of cheese consumption shows a higher slope in the 1st and 2nd tertiles from 1996 to 2004. Additionally, the decreasing slope of consumption since 2014 in the 1st tertile is lower compared to the 3rd tertile. The gap between the 1st and 3rd tertiles of households’ expenses has decreased (from 2.09 kg in 1991 to 1.17 kg in 2021).

Figure 4, illustrates an overall increasing trend in dairy consumption across all tertiles until 2005, followed by a decrease with two sharp drops. The first drop occurred in all tertiles since 2011, with the most significant decrease in the 3rd tertile. The second drop was observed around 2018, which had a steeper slope in the 2nd and 3rd tertiles. For instance, the total dairy consumption for the 1st tertile decreased from 59.64 kg in 1991 to 49.04 kg in 2021, while the total dairy consumption for the 3rd tertile decreased from 84.88 kg in 1991 to 81.74 kg in 2021. The weighted mean of *per capita* total dairy consumption increased from 63.39 in 1991 to 68.34 kg in 2021.

**Figure 5:**
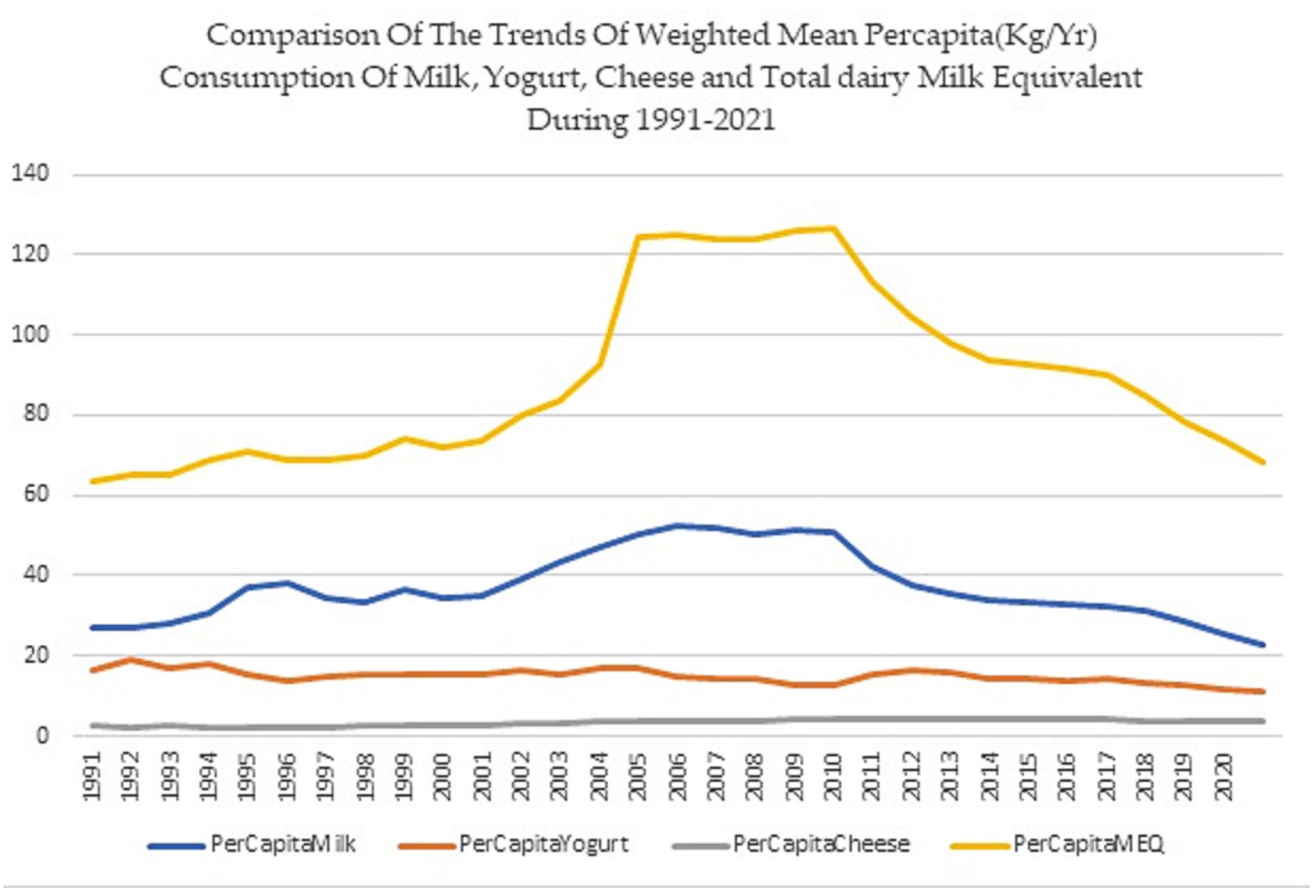
Comparison of the trends of weighted mean *per capita* consumption of milk, yogurt, cheese and total dairy based on milk equivalent during 1991–2021.

As shown in Figure 5, the mean *per capita* consumption of milk and yogurt has decreased and cheese has increased over time, from 26.77 kg, 16.63 kg, and 2.42 kg in 1991 to 22.68 kg, 11.06 kg, and 3.79 kg in 2021, respectively. It is also apparent that up to 2015, yogurt has been a substitute for milk in most years, and its consumption increased while milk consumption decreased. However, since then, yogurt consumption has also been on a decreasing trend, similar to milk. On the other hand, cheese consumption shows a similar trend to milk, except that, unlike milk, its overall trend is still increasing.

Findings of the estimated demand function for milk, yogurt, cheese, and total dairy consumption based on milk equivalent are presented in Table 3. This highlights that household expense was positively associated with the amount of all types of dairy consumption, while product price had a negative association. Family size showed a positive relationship between yogurt and total dairy consumption but a negative relationship with cheese consumption. Family education score had a significant positive relationship with milk, but a negative association with cheese and total dairy consumption. Housewives’ job scores showed a statistically significant positive relationship with milk and cheese consumption. The share of families with children under 5 years old had a significant negative relationship with all dairy items, but not with total dairy, while the share of families with older adults members (over 60 years old) had an inverse relationship with yogurt and cheese, and total dairy consumption. Living in urban areas was negatively associated with milk consumption but positively associated with cheese and total dairy consumption.

**Table 3:** Results of estimating milk, yogurt, cheese, and total dairy demand function.

	Milk		Yogurt		Cheese		Total dairy (Milk equivalent)	
	*β*	*t*	*β*	*t*	*Β*	*t*	*β*	*t*
Family size	−1.873	−1.360	2.345***	3.740	−0.422***	−3.530	15.07**	2.340
Family education score	1.452*	2.030	−0.355	−0.850	−0.176**	−2.080	−7.22**	−2.190
Housewife job score	12.607***	9.060	−0.466	−0.940	0.297**	3.100	−27.89**	−2.630
Share of pasteurized milk	47.167***	16.270						
Household expense	6.83E-09*	1.870	4.54E-09**	2.590	2.14E-09***	6.270	2.58E-08*	1.760
Product price	−0.051***	−6.140	−0.001*	1.860	−1.23E-04**	−2.880	−2.46E-04**	−2.120
Share of women-headed households	−60.036**	−4.130			9.754***	8.520	−76.366**	−5.10
Share of families with children under 5 yr	−14.267**	−4.910	−7.282**	−3.130	−3.966***	−8.120	−28.79	−0.860
Share of families with members more than 60 yr	3.247	0.140	−19.307***	−4.170	−11.303***	−8.550	−223.43**	−2.450
Urban living	−55.220***	−5.890	2.924	0.920	1.176 *	1.900	80.98*	1.850
Intercepts	41.604	3.170	10.167*	1.770	7.226***	6.630	87.198*	1.850
F	237.220**		41.26**		−0.639**		47.99**	
*R* ^2^	0.891		0.531		0.865		0.012	
F- limer	5.17	*p*-value: 0.00	6.720***	*p*-value: 0.000	4.540***	*p*-value: 0.000	13.01***	*p*-value: 0.001
Hausman test	43.05	*p*-value: 0.00	51.310***	*p*-value: 0.000	33.040***	*p*-value: 0.000	259.69***	*p*-value: 0.089

*, **, *** are significance levels of 90, 95, and 99 percent, respectively. Results of the Fixed effect model for milk, yogurt, and cheese and the random effect model for Total dairy are presented.

## 4 Discussion

The present study showed that despite an increasing trend in dairy consumption from 1997 to 2005 overall dairy consumption among Iranian households had a declining trend, with two significant drops in 2011 and 2019. There is also obvious that all tertiles have decreased their consumption since 2007, but 2nd and 3rd tertiles are less affected at least till 2018.

**Figure 6:**
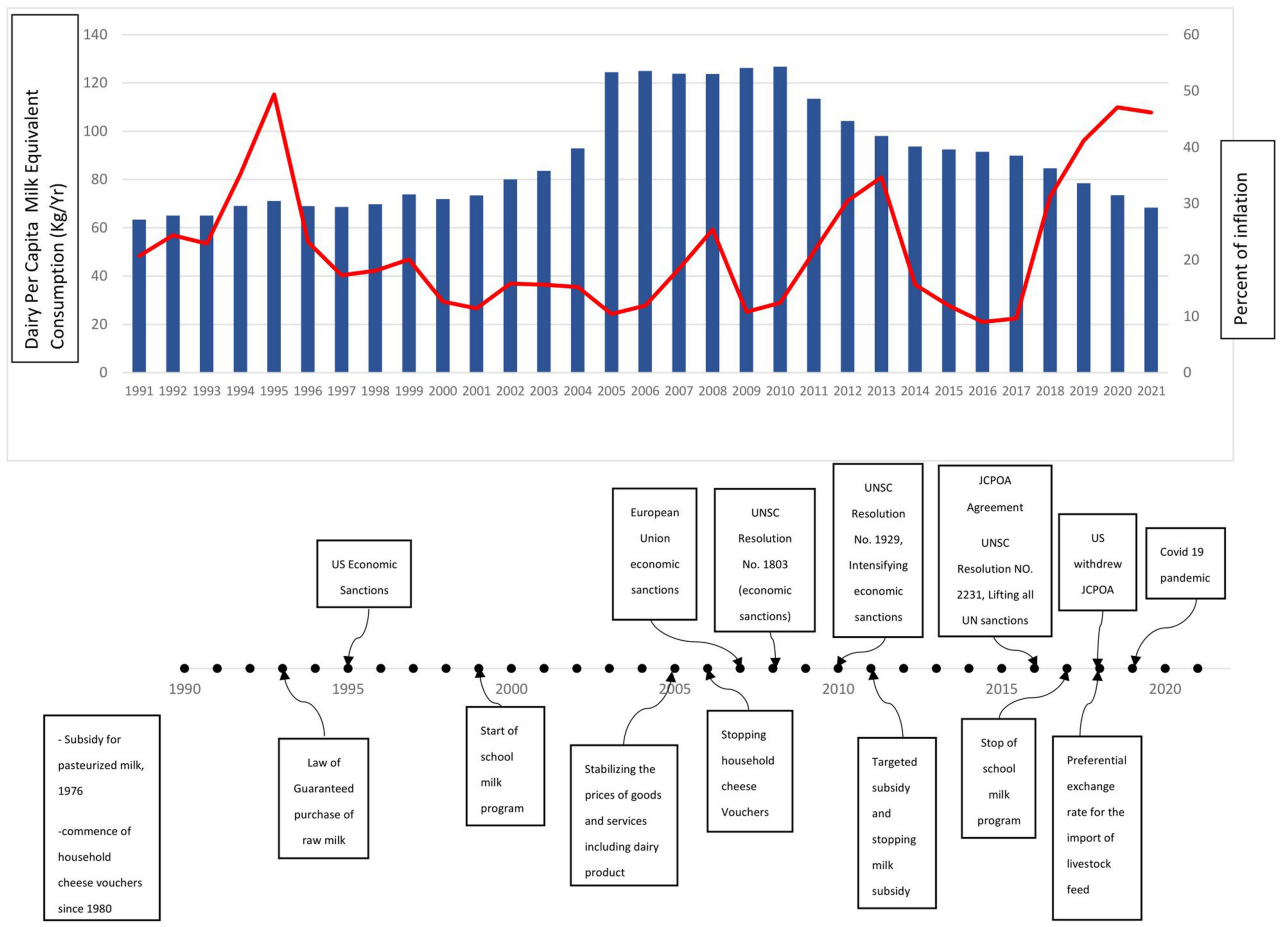
Total dairy consumption trend based on milk equivalent in comparison with inflation trend and timeline of major dairy food system and contextual events.

As shown in Figure 6, Iran began subsidizing pasteurized milk in (33). After the revolution, with the start of the Iraq war, the government introduced a voucher system to provide some main food items such as cheese. By the end of the war, due to the adoption of expansionary policies for the prosperity of production, the inflation rate increased and reached a peak of 50% (34). Despite the US imposing economic sanctions at that time (35, 36), the inflation rate began to decline. Meanwhile, the government implemented policies such as the guaranteed purchase of raw milk (37) and the school milk program (38) to safeguard producers and consumers, contributing to an uptrend in dairy consumption until 2005. However, with the change of government in 2005 and Iran’s nuclear program’s escalation, leading to its referral to the Security Council in 2006 inflation and sanctions intensified (34). The government implemented price stabilization measures (39), to counter inflation’s effects while termination of the household cheese voucher program. But a slight reduction in dairy consumption is obvious. In 2010, the United Nations Security Council (UNSC) approved resolution number (40), further intensifying previous sanctions. Meanwhile, at the beginning of 2011, following government economic reforms and the Targeted Subsidies Law (41), subsidies were removed for goods such as gasoline, oil, water, and milk, substantially increasing milk and dairy prices during five years, and the inflation rate starts its ascending trend. The government distributed some of its revenue to the public as cash transfers to combat price hikes, but this led to significant inflation. This is the first considerable drop in dairy consumption. The trend of declining dairy consumption slowed after 2014, coinciding with a decrease in inflation rates and the JCPOA’s approval in 2015. However, the US’s withdrawal from the JCPOA in 2018, coupled with the COVID-19 pandemic in 2019, led to a second drop in dairy consumption due to an increase in inflation rates. Despite the government’s efforts to control dairy prices by granting preferential exchange rates for livestock feed imports, dairy consumption continued to decline.

Other cross-sectional and longitudinal studies also mention the low consumption of milk and dairy products among Iranians and its decreasing trend (42–44) despite the increase in dairy production (45). For example, in a cross-sectional study on a subsample of a population-based cohort study of the “Tehran Lipid and Glucose Study” with 827 subjects (357 men and 470 women) aged 18–74 years, the mean (±SD) consumption of milk, yogurt, and cheese was 0.7 ± 0.2, 1.06 ± 0.6, and 0.9 ± 0.3 servings/day, respectively (46). The Iran National Comprehensive Study on Household Food Consumption Pattern and Nutritional Status 2001–2003 also showed that the *per capita* means daily consumption of milk and dairy groups in urban and rural households was 142.3 ± 1.8 and 134.1 ± 2.7 grams/day, respectively, with a total of 139.6 ± 1.5 grams/day (around 51 kg/year) (11) which is lower than the amount reported in the present study at that. This could be because the Household Expenditure and Income Survey used in the present study is a reliable proxy for household food consumption but does not consider food loss, waste, or food used for other reasons such as guests (19).

The findings of the study also showed that household expenses (as a proxy of income) have a positive relation and food price shows a significant inverse relation with milk, yogurt, and cheese consumption, although, among them, cheese seems less affected while milk was the most affected dairy items. Additionally, these studies show higher amounts of yogurt consumption, while milk consumption purchase is higher in the present study. This is probably due to the culture of home yogurt making, mainly in rural areas, e.g., in Turkey, where consumers of non-pasteurized milk, buy milk to make yogurt (47).

Iran’s economic sanctions have resulted in austerity policies causing inflation, stagnation, and recession, resulting in lower household incomes and rising commodity prices, and likely leading to a sharp decline in dairy consumption (17). Targeting subsidies also led to a significant increase in milk and dairy prices, as production costs rose due to the elimination of subsidies as well as removing milk subsidy and high inflation due to the adoption of this policy. From 2010 to 2014, the price of milk increased by approximately four times based on the author’s calculation.

As mentioned before, inflation reduces household real income, while studies indicate that economic factors such as price and income affect dairy consumption (48–51). For example, Maitah and Smutka noted an increase of about 68% in milk consumption in the Middle East and North Africa during 1990–2008, with country- level *per capita* income being the most significant factor for this rise (52). Bousbia identifies income level and price as major factors impacting the variation of dairy product consumption (53). In Brazil, the cost of dairy products has been an important factor related to their low intake (54). It is worth noting that all income groups are not affected by increasing the price or inflation in the same value. Low-income groups are more sensitive to income and price fluctuations as allocate a larger share of their income to food than higher-income households (55, 56). On the other hand, low consumption levels respondents are more responsive to changes in income (57). As household income increases, the possibility of consuming dairy products increases. In other words, a more diversified diet using more expensive animal food sources like milk and dairy products is possible with increasing household income or decreasing the prices of these products.

However, the effect of income is not uniform on all dairy products. The findings show that although cheese consumption (mainly white cheese) has a slower decreasing trend, its overall trend indicates an increase. Most Iranians consume cheese with bread for breakfast, making cheese consumption a part of their food culture (58). This could justify the slower decreasing trend, and the study’s findings align with those of other studies (58, 25). In 2019, another drop in dairy consumption was observed, mainly in the 2nd and 3rd tertiles of income, which could be attributed to the COVID-19 pandemic and high inflation rates of 31.2, 41.2, and 47.1% during 2019–2021. A nationwide cross-sectional survey by Nikooyeh et al. found a significant decrease in dairy product consumption, particularly milk, and yogurt, in a high proportion of households during the COVID-19 epidemic lockdown (58). A similar decreasing trend in dairy consumption due to COVID-19 has also been reported for other countries such as China mainly due to the price increases, income loss, and insufficient savings combination (59). However, some others reported increasing dairy products consumption in the same period compared with that before the COVID-19 outbreak due to consumer health concerns (60).

The present study demonstrated that factors such as household composition and size have an impact on total dairy consumption. It seems that larger families tend to prefer yogurt consumption to cheese. Yekta et al., reported that household size has a positive and statistically important effect on yogurt consumption and large families consume more yogurt than small families. Other studies have also found a positive association between household size and yogurt consumption (47). It seems that family size exerts its effect on dairy consumption in two ways. As Phuong et al. (61) concluded, family size has a positive effect on milk product buying decisions, as families perceive dairy as part of their children’s diet (62); however, it has a negative effect on household’s dairy expenditure on a *per capita* basis. Radam et al., also showed that consumers from households with four or more people, including children, tended to be more price-sensitive and less likely to pay for expensive food (63). Hagh et al., found that households in the lowest-income groups consumed significantly lower amounts of livestock and dairy food items as the family size increased (64). Also, Hannan et al. reported that as the family size increases, the budget shares for milk will decrease (65). Based on the Iranian cultural preference for yogurt consumption (66) and the perceived health benefits associated with yogurt, it can be concluded that family size has a significant impact on dairy consumption, particularly yogurt.

Family education score was negatively related to yogurt, cheese and total dairy consumption. Vakili et al., also showed that People with a high school diploma and lower levels of education consumed substantially more dairy products than more educated ones (67). Contrary to these findings, some other cross-sectional studies in Iran (66), indicated that higher education level increases the probability of choosing dairy products. Streeter and colleagues also showed that in China, better-educated respondents favor lower-calorie diets with more animal foods, fruit, and dairy but less cereals (57). Similar results were observed in Turkey where dairy product consumption was higher in those with a higher level of education (68). In recent years, there has been an international discourse about the potential association between high dairy consumption and an increased risk of certain non-communicable diseases (69, 70). As educated individuals often have greater access to information (71), they may be more aware of such concerns, which could have influenced their consumption patterns. Furthermore, there has been significant negative publicity surrounding the Iranian processed dairy industry, such as the unauthorized use of palm oil by the dairy industry being brought to the media’s attention by the Minister of Health in 2015, or the discussion on aflatoxin contamination of milk on a national television show by an expert. However, it is important to note that there is also evidence highlighting safety concerns related to dairy consumption (72), as well. Considering that individuals with higher education often exhibit more sustainable and conscious eating behaviors (73), these factors may influence their dairy consumption.

Our study revealed a positive correlation between the job score of mothers and consumption of milk and cheese, while showed a negative correlation with overall dairy consumption. These findings align with the research conducted by Kaheni et al., who also demonstrated a positive relationship (*p* = 0.03) between maternal employment and milk consumption among children aged 6–11 in Birjand (74), Iran. Another study by Widodo et al. (75) reported higher dairy consumption in Indonesian children whose mothers had permanent jobs (75). However, these findings contrast with Afrin et al.’s systematic review, which indicated that maternal employment tends to have more adverse effects than favorable on children’s dietary patterns. The review concluded that in dual-income families, maternal employment may provide the financial means to afford healthier food options. Nevertheless, it also highlighted that employment can impose time constraints that affect meal-related behaviors (76). Therefore, the increased consumption of milk and cheese observed in our study could be attributed to greater resources provided by working women. However, the lower overall dairy consumption might be a result of time constraints, reduced supervision, and less caregiving by these women toward their families and possibly themselves. Interestingly, our study also revealed that female-headed households tend to consume more cheese and total dairy while have lower milk consumption. This finding is in contrast with other studies that have shown female-headed households have lower dairy consumption (58, 77) probably due to their limited resources. However, our study also found a positive relationship between female-headed households and cheese consumption. This may be due to the role of cheese in Iranian food culture, where cheese and bread can serve as a meal and may also be seen as a valuable source of protein for the households. Due to cheese’s substantial weight in converting to milk equivalent, it is probable that it also influences overall dairy consumption.

We found a significant negative relationship with having under 5 age children and all dairy items but not total dairy. In contrast to the present study, several studies indicated that the presence of young children in the household positively affects household dairy consumption (78–81). Radam et al. showed that households with children less than 12 years of age were generally less concerned about price and more interested in purchasing safe and wholesome food (63). It may be rooted in economic issues as studies show there is a well-established relationship between household poverty and having children under the age of five. In general, households living in poverty are more likely to have young children. This is because poverty is often associated with a lack of access to education, healthcare, and family planning services, which can lead to higher fertility rates (82, 83).

The present study demonstrated that the presence of family members over 60 years in households was also inversely related to yogurt, cheese and total dairy consumption. In contrast, Possa et al. (54) showed that older adults subjects had the highest total dairy intake, milk, and cheese in Brazil (54). In another study on global, national, and regional estimates of milk and dairy beverage consumption, higher values were reported for the older adults (84). Align with the present study, a study in Switzerland reported that adults and the older adults did not consume the recommended number of dairy products per day. Besides, 25.0% of the individuals reduced dairy consumption to decrease fat or cholesterol intake (85). Although the amount of consumption is much less than in this study in Iran, geriatric health concerns may also affect older adults dairy consumption. For example, traditional Iranian medicine considers dairy food as cold temperament foods that exacerbate bone and joint pains that should be avoided (86). On the other hand, medical costs, in particular, can be a significant burden for older adults, particularly those who have chronic health conditions or require long-term care which may result in inappropriate economic conditions which affect their consumption as well (87).

The present study showed that residing in urban areas was negatively related to milk and positively related to cheese and total dairy consumption. In contrast with these findings, several studies showed that dairy product consumption among urban households is much more than in rural ones (57, 61, 79, 88). The difference is due to the different methodologies. In other words, these studies evaluate consumption while Household Expenditure and Income Survey studies assess household purchases. It seems that in rural areas people mainly buy milk and using home processing convert it to dairy products while urban people purchase less milk and instead, buy and consume more cheese. This difference may be attributed to rural households’ access to raw milk as well as market and dairy products. A study in rural Ethiopia demonstrated that household cow ownership increases children’s milk consumption and this ownership is less important where there is good access to local markets (89). Moreover, regarding the urban–rural income gap (90, 91), the economic situation may also negatively affect cheese consumption among rural households (77).

Strengths of the current study included the use of panel data to consider crosses effects (expenditure deciles) in addition to time series to draw a picture of Iranian dairy consumption using recent and large nationally representative data. Assessing the role of demographic and socio-economic factors in dairy consumption allows for deep problem identification and adoption of appropriate policy interventions unique to these households. It is also important to consider the limitations of the Household Expenditure and Income Survey analysis. One of the most important is the lack of information about the food allocation within the family. Also, the present study did not consider other determinants of dairy consumption such as the role of habits, nutritional knowledge, perceptions, and hedonic attributes of dairy products.

## 5 Conclusion

In conclusion, this study provides insight into the trends and determinants of dairy consumption in Iranian households. It also showed that the determinants of consumption vary for different dairy items, and it is more appropriate to evaluate them individually rather than as a group. This is particularly evident when converting dairy to milk equivalent, as the consumption pattern sometimes follows the trend of cheese due to its high weight in the conversion process. Therefore, using milk equivalent as a method to evaluate overall dairy consumption may not be a perfect approach. The findings indicate a declining trend in overall dairy consumption, with household income and product price showing significant positive and inverse relationships with milk, yogurt, and cheese consumption. Family size, family education score, and head of the HH spouse’s job score were found to be positively correlated with dairy consumption, while the presence of children under the age of five and older adult members (over the age of 60) had inverse relationships with yogurt and cheese consumption. Female-headed households tend to purchase more cheese, while their milk consumption is significantly lower. Residing in urban areas was negatively related to milk consumption, while cheese consumption was higher in urban areas. Based on the findings, it is necessary for policymakers to adopt appropriate economic policies at the macro level and take necessary measures to improve the economic situation of the society. Additionally, implementing supportive policies and allocating subsidies to promote dairy consumption can be achieved through interventions such as food support programs for female-headed households or the older adults. Furthermore, implementing suitable educational programs at the community level, along with supportive programs to enhance knowledge and attitudes toward dairy consumption, can also help to improve the consumption of this food group. However, further research is needed to explore the role of other determinants of dairy consumption, such as dietary habits, nutritional knowledge, and perceptions of dairy products.

## Data availability statement

The data presented in this study is not publicly available due to privacy restrictions. Requests to view these datasets should be directed to the corresponding author.

## Author contributions

RR: Conceptualization, Data curation, Formal analysis, Investigation, Methodology, Project administration, Software, Writing – original draft, Writing – review & editing. HE-Z: Conceptualization, Methodology, Supervision, Writing – review & editing. DG: Data curation, Formal analysis, Methodology, Writing – review & editing. EM: Data curation, Formal analysis, Investigation, Software, Writing – review & editing. NO: Formal analysis, Writing – review & editing. HH: Formal analysis, Writing – review & editing. SH: Data curation, Software, Writing – review & editing. HR: Conceptualization, Methodology, Supervision, Writing – review & editing.
