# Supplementary material for: A 30-year trend of dairy consumption and its determinants among income groups in Iranian households
Source: Front Public Health. 2024 Feb 15;12:1261293. doi: 10.3389/fpubh.2024.1261293 (PMC10903262; doi:10.3389/fpubh.2024.1261293)
Supplement: Supplementary file 1 [file Table_1.docx]

**Supplementary Material**

Table 1: Summary of Selected Studies Investigating Individual Factors Affecting Dairy Consumption.

| Author(s) and Year | Objective | Methods | Main Findings |
| --- | --- | --- | --- |
| Heady (2023)[1] | To investigates the economic factors that contribute to the differences in dairy consumption among young children across countries. | The study utilizes data from the Demographic and Health Surveys (DHS) sample, which includes 114,560 children from 53 low- and middle-income countries (LMICs) during the period of 2008-2016. | variations in wealth levels across LMICs are the most significant predictor of differences in dairy consumption, followed by disparities in dairy prices. Additionally, the availability of refrigeration is found to be a predictor of dairy consumption, while water quality has an impact on powdered milk consumption. |
| Vakili et al (2023)[2] | To examine the factors associated with the rate of dairy product intake, focusing on social health-related beliefs and predictors of dairy consumption based on the transtheoretical model. | The study involved surveying 981 subjects randomly selected from the citizens of Mashhad, Iran, in 2014. The data collection included demographic surveys and questionnaires administered in various public places. | The findings reveal a significant relationship between dairy consumption and gender (P < 0.001). Additionally, factors such as age, educational level, job status, and opium addiction were found to be significantly associated with the status of dairy consumption. |
| ILIE et. al (2021)[3] | Aimed to identify the main factors influencing consumers' choices when selecting milk or dairy products and to determine if these factors are shifting towards including sustainability-related aspects. | The researchers conducted a quantitative survey using a structured investigation technique, employing a self-administered questionnaire. | The results indicate that while price and store availability continue to be important criteria in the decision-making process, respondents are also considering new aspects that are potentially linked to sustainable behavior. These include ecological certification, country of origin, and traditional products. However, the consideration of these factors depends on the respondents' income level, with higher incomes enabling individuals to take these new criteria into account. |
| Ortez et.al (2021)[4] | To investigate the dairy buying behavior of households in the United States, with a specific focus on households that frequently purchased food for children and whether their dairy buying behavior differed | The researchers employed an online survey to collect demographic information and data on dairy product purchasing behavior from 1440 US residents from other households. | The findings revealed that households that frequently bought food for children tended to purchase larger quantities of fluid milk, with a preference for higher fat content. Additionally, these households purchased yogurt more frequently compared to other households. When examining labeling information, price was found to be of the highest interest to respondents, followed by the expiration date, particularly for meat, eggs, or milk product packaging. |
| Haq et. al (2020)[5] | To examine the differences in food intake among rural families, taking into account various income levels and family sizes. Also, to analyze the barriers faced by rural families in increasing their consumption of diverse food items. | The study utilized a quantitative survey with a questionnaire administered to 200 rural households in the Punjab Province of Pakistan. | The findings revealed that both low and high livelihood diversified families with low income and large family size tended to consume less expensive food items. On the other hand, regular consumption of fruits, bakery products, and livestock and dairy items was strongly associated with higher income and smaller family size. |
| French et al. (2019)[6] | To investigate the relationship between household income and the quality of household food purchases. | The researchers collected food purchase receipt data over a period of 14 days from 202 urban households participating in a study on food shopping. The data was then scored using the Healthy Eating Index 2010, which assesses the nutritional quality of food purchases | After adjusting for factors such as education, marital status, and race, the researchers found that higher income households had significantly higher dairy scores compared to lower income households. The mean dairy score for higher income households was 5.6 (sd = 3.0), while it was 5.0 (sd = 3.3) for lower income households (p = .05). This suggests that higher income households tended to have better quality dairy purchases compared to lower income households. |
| Ahmadi Kaliji et. al. (2019)[7] | To examine consumers' preferences for dairy products and identify the factors that influence their choices. | The researchers conducted a survey with 275 participants in Sari, Iran in 2018, utilizing questionnaires for data collection. The nested logit model was employed for the analysis of the data. | The findings revealed several factors that influenced the choice of dairy products. Price and family cost were found to decrease the probability of selecting certain products. On the other hand, variables such as age, education level, and attention to exercise were associated with an increased probability of choosing dairy products. This suggests that younger individuals, those with higher education levels, and those who prioritize exercise are more likely to select dairy products. |
| Bousbia et. al (2017)[8] | Aimed to analyze consumer behavior regarding milk and dairy products and explore the potential effects of various variables on consumer decision-making when purchasing these products. | The researchers conducted a survey, utilizing questionnaires, to collect data on milk consumption, including perceptions before purchase and actual consumption. The survey was conducted among 326 randomly selected households located in urban, semi-urban, and rural areas. | The findings of the study highlighted several significant socio-economic variables that explained individual differences in consumer behavior. These variables included taste, trust, health benefits, packaging, type of shop, brand, origin of the product, and advertising. These factors played a crucial role in shaping consumer preferences and choices when it came to milk and dairy products. |
| Possa et. al (2017)[9] | To estimate the consumption of dairy products in the Brazilian population. | Data from two non-consecutive food records from 34,003 individuals in the first Brazilian nationally representative dietary survey (2008-2009) were used to estimate the dairy products intake and its determinants. | The prevalence of consumption was higher among females, elderly, residents of the South region, and among individuals from higher per capita monthly family income.  Furthermore, the study identified four major factors that contributed to the variation in dairy product consumption. The geographic area accounted for the largest variation at 43.98%, followed by the number of children per household at 20.80%, income level at 20.76%, and price at 9.17%. |
| Streeter (2017)[10] | The socioeconomic determinants of food and macronutrient consumption in China over the past two decades were examined | The analysis utilized panel regression techniques and encompassed data from the period between 1989 and 2009. | he study's findings demonstrated that as income levels rose, the consumption of animal foods, fresh fruit, and dairy products increased, while cereal consumption decreased. Individuals with lower initial consumption levels were more susceptible to income changes, indicating a greater influence on their food choices.  Furthermore, the study revealed that individuals with higher levels of education tended to opt for lower-calorie diets, which included a higher proportion of animal foods, fruit, and dairy products, but a lower proportion of cereals.  Lastly, the study observed that urban residents had higher consumption levels of animal foods, fresh fruit, and dairy products compared to their rural counterparts. |
| Widodo et al (2016)[11] | This study aimed to investigate the relationships between nutrient intake, dairy consumption, and socioeconomic variables. | Data on food consumption, including 24-hour recall, as well as information on the frequency of dairy consumption, anthropometric measurements, and sociodemographic variables were collected from a sample of 3600 children aged 0.5 to 12 years old. | The findings of the study indicated that there was no difference in dairy consumption between boys and girls, but it was found to be higher in urban areas. Additionally, dairy consumption was positively correlated with the education level of the mother, the employment status of the mother (permanent job), and the wealth status of the family, particularly in the upper levels. |
| Yekta et.al. (2015)[12] | Aimed to investigate the consumer and household characteristics that influence the consumption decision of yogurt. | They collected cross-sectional survey data from 8549 households in Turkey. | The findings of the study indicated that several factors had a statistically significant impact on yogurt consumption. These factors included household income, household size, education level, age, working status, gender of the household head, and residential areas (rural or urban).  The results suggest that higher household income and smaller household size were associated with increased yogurt consumption. Additionally, individuals with higher education levels, younger age groups, and working individuals were more likely to consume yogurt. The gender of the household head and the rural or urban residence also played a significant role in yogurt consumption decisions. |
| Phoung et. al (2015)[13] | Aimed to analyze the effects of socio-economic and demographic variables on Vietnamese households' decision to purchase dairy products and the amount spent per capita on these items. | The data used for the analysis was obtained from the Vietnamese Household Living Standard Survey in 2010. | The results of the study indicated that, apart from income, certain household characteristics had a significant and positive impact on dairy product expenditure. Specifically, being an urban household, having a female head of household, and having children in the household were found to influence the decision to spend on dairy products.  These findings suggest that urban households, female-headed households, and households with children are more likely to allocate a higher portion of their expenditure towards dairy products in Vietnam. |
| Singh et al (2015)[14] | aimed to quantify the global, regional, and national levels of sugar-sweetened beverages (SSB), fruit juice, and milk intake among adults aged 20 and above in the year 2010. | the researchers identified, obtained, and assessed data on the consumption of these beverages in adults, categorized by age and sex. They collected information from 193 nationally- or sub nationally-representative diet surveys conducted worldwide. Additionally, data on the availability of milk, fruit juice, and SSB in 187 countries were extracted from annual food balance information provided by the United Nations Food and Agriculture Organization. | The findings of the study revealed that milk intake was highest among older adults. |
| Ebru Onurlubaş and Neslihan Yılmaz (2013)[15] | The factors influencing the consumption habits of pre-packaged and fresh milk in families residing in the Keşan township of Edirne were examined. | The data utilized in the study were collected through face-to-face surveys conducted with 166 families. The researchers analyzed the relationship between various factors and milk consumption habits using chi-square tests. | The findings indicated that there was a significant relationship between the age and average monthly income of the families and their consumption of pre-packaged milk. As the income level of the families increased, there was an increase in the consumption of pre-packaged milk. However, no significant relationship was found between gender, level of education, and the consumption of pre-packaged milk based on the chi-square test results.  Furthermore, the chi-square analysis revealed a significant relationship between age and the consumption of fruit milk. As the age of individuals increased, the consumption of fruit milk decreased. |
| Maitah, M. & Smutka, L. (2012)[16] | Aimed to analyze the factors that determine the supply and demand for liquid milk and identify the main problems and constraints hindering milk production. | The research approach involved a descriptive analysis to introduce the study problem, as well as the application of quantitative analysis methods, including linear regression analysis. These methods were used to examine the variables relevant to the study, based on the available data. | The findings of the study indicated that population, per capita income, and the quantity of milk produced were the most important factors. The results revealed that a 1% increase in population, per capita income, and the quantity of milk produced corresponded to an increase in milk consumption by 1.3%, 2.86%, and 0.611%, respectively. |
| Hannan et. al (2010)[17] | The objective was to examine the factors that influence the household demand for dairy products in Bangladesh. | The study utilized data from the Household Income and Expenditure Survey (HIES-2000), which included a comprehensive set of micro-level cross-sectional data from 7,440 households. This dataset was originally published by the Bangladesh Bureau of Statistics (BBS-2002). | The findings of the study revealed that family size and the occupation of the household head had a statistically significant impact on household demand behavior for dairy products. These factors were identified as key determinants affecting the demand patterns and preferences of households in relation to dairy products. |
| De Alwis et. al (2009)[18] | Aimed to analyze the consumer attitudes, demographic, and economic factors that influence fresh milk consumption. | The data for the study was collected through a consumer survey conducted in the Kandy district, where randomly selected supermarkets, groceries, milk bars, and retail shops were included. | The study revealed that household monthly income, health problems affecting fresh milk consumption, and the level of education played a crucial role in milk consumption. |
|  | analyzing the consumer attitudes, demographic and economic factors that affecting fresh milk consumption among the mid-country consumers of Sri Lanka | a consumer survey conducted in Kandy district at randomly selected super markets, groceries, milk bars and retail shops | age of the respondent, cost and usage related attitudinal factor and Nutrition related attitudinal factors are the key determinants of milk consumption levels; however, household monthly income, health problems affect on fresh milk consumption and level of education play a more important role in consumption |

1. Headey, D., *Can dairy help solve the malnutrition crisis in developing countries? An economic analysis.* Animal Frontiers, 2023. **13**(1): p. 7-16.

2. Vakili, V., et al., *Effect of Social Beliefs on Consumption of Dairy Products and Its Predicting Factors Based on the Transtheoretical Model: A Population-Based Study.* Journal of Environmental and Public Health, 2023. **2023**: p. 5490068.

3. Ilie, D.M., et al., *Consumer Choice for Milk and Dairy in Romania: Does Income Really Have an Influence?* Sustainability, 2021. **13**(21): p. 12204.

4. Ortez, M., et al., *Dairy product purchasing in households with and without children.* JDS Communications, 2021. **2**(1): p. 7-12.

5. Haq, S., et al., *Rural families food intake behaviour in relation to livelihood diversification, income differences and family size.* International Journal of Consumer Studies, 2020.

6. French, S.A., et al., *Nutrition quality of food purchases varies by household income: the SHoPPER study.* BMC Public Health, 2019. **19**(1): p. 231.

7. Ahmadi-Kaliji, S., et al., *Factors affecting consumers' dairy products preferences.* AGRIS on-line Papers in Economics and Informatics, 2019. **11**(2): p. 3-11.

8. Aissam Bousbia, et al., *Analysis of Factors Affecting Consumer Behavior of Dairy Products in Algeria: A Case Study from the Region of Guelma.* International Journal of Agricultural Research., 2017. **12**: p. 93-101.

9. Possa, G., et al., *Dairy products consumption in Brazil is associated with socioeconomic and demographic factors: Results from the National Dietary Survey 2008-2009.* Revista de Nutrição, 2017. **30**(1): p. 79-90.

10. Streeter, J.L., *Socioeconomic Factors Affecting Food Consumption and Nutrition in China: Empirical Evidence During the 1989–2009 Period.* The Chinese Economy, 2017. **50**(3): p. 168-192.

11. Widodo, Y., et al., *The effect of socio-demographic variables and dairy use on the intake of essential macro- and micronutrients in 0.5-12-year-old Indonesian children.* Asia Pacific Journal of Clinical Nutrition, 2016. **25**(2): p. 356-367.

12. Yekta, G. and A. Cuma, *An Analysis of Household¡¯s Yogurt Consumption in Turkey.* Journal of Food and Nutrition Research, 2015. **3**(4): p. 285-289.

13. Van Phuong, N., T.H. Cuong, and M. Mergenthaler, *Effects of household characteristics on expenditure for dairy products in Vietnam.* International journal of Research Studies in Agricultural Sciences, 2015: p. 1-13.

14. Singh, G.M., et al., *Global, regional, and national consumption of sugar-sweetened beverages, fruit juices, and milk: a systematic assessment of beverage intake in 187 countries.* PloS one, 2015. **10**(8).

15. Onurlubaş, E. and N. Yılmaz, *The factors affecting milk consumption preferences of the consumers in Edirne Keşan township.* Journal of Food, Agriculture & Environment, 2013. **11**(3&4): p. 516-518.

16. Maitah, M. and L. Smutka, *Economic analysis of milk production and consumption in the Middle East and North Africa.* Acta Universitatis Agriculturae et Silviculturae Mendelianae Brunensis, 2012. **60**: p. 245-254.

17. Hannan, M., A. Dutta, and H. Kabir, *Household demand for dairy products in Bangladesh: An Application of AIDS Model.* Journal of the Bangladesh Agricultural University, 2010. **8**(1): p. 121-126.

18. De Alwis, A., J. Edirisinghe, and A. Athauda, *Analysis of factors affecting fresh milk consumption among the mid-country consumers.* Tropical agricultural research and extension, 2011. **12**(2).
